# High impact polytriazole resins for advanced composites

**DOI:** 10.1080/15685551.2020.1761584

**Published:** 2020-05-05

**Authors:** Mingming Ma, Xiuyun Wang, Zhuoer Yu, Liqiang Wan, Farong Huang

**Affiliations:** a Key Laboratory of Specially Functional Polymeric Materials and Related Technology of the Ministry of Education, East China University of Science and Technology, Shanghai, China; b Research & Development Center, Xi’an Aerospace Composites Research Institute, Xi’an, China

**Keywords:** ‘Click’ polymerization, polytriazole resin based on polyether, high performance resin, resin with low curing temperature, mechanical properties

## Abstract

Three azido-terminated poly(ethylene glycol) macromonomers (ATPEGs) were synthesized from poly(ethylene glycol)s (PEGs) and characterized. The extended polytriazole (EPTA) resins were prepared from the macromonomers, azide and alkyne monomers. Toughening effect of PEGs on polytriazole resins was analyzed by means of mechanical, thermal and electronic microscope characterization. The results show that molecular weight and content of ATPEGs have great influence on the thermal and mechanical properties of cured EPTA resins. The impact strength of cured EPTA resins increases with the increase of the amount and molecular weight of ATPEGs. The flexural strength and heat resistance of cured EPTA resins decrease with the increase of addition amount and molecular weight of ATPEGs. High impact EPTA resins were obtained.

## Introduction

In 1893, Michael [1] first discovered the 1,3-dipolar cycloaddition reaction of alkynyl and azide which resulted in the formation of triazole ring compounds. Sharpless group [2] synthesized highly regioselective 1,4-substituted triazole polymers using copper (I) as a catalyst through the ‘Click’ reaction of alkyne and azide compounds. The ‘Click’ reaction has good designability and effectiveness, and can be used to prepare a variety of triazole polymers with different structures [3–15].

Huang group [16–25] has prepared a series of thermosetting polytriazole resins by 1, 3-dipolar cycloaddition reaction of azides and alkynes since 2002. The study results have shown that the polytriazole resins are cured at low temperature (60 ~ 80 °C) and the cured resins have good thermal properties and mechanical properties, and the resins have been used as matrices in advanced composites. Polytriazoles have also been found in applications as elastomers, adhesives, membranes, etc. Ragin, et al. [26] synthesized a shape memory elastomer (E-SMP) by using copper catalyzed azide-alkyne cycloaddition reaction. The E-SMP exhibited fast shape recovery (12 s) and is thermally stable up to 260 °C. Tang, et al. [27,28] introduced a facile approach to fabricate automatically programmable SMPs (AP‐SMPs) based on bilayer structures of poly(1,2,3‐triazolium) vitrimers (VPTAs), which are derived from poly(1,2,3‐triazole) with dynamic covalent crosslinks, greatly increasing the designability of SMPs. Tang, et al. [29] successfully synthesized hyperbranched polytriazoles with excellent adhesive properties on copper, aluminum and iron. Stefan, et al. [30] reported the synthesis of organic solvent resistant polytriazole membranes using a sustainable process and investigated the mechanical properties by measuring the creep recovery.

Recently, advanced polymeric composites require the resins with high toughness to meet the development of high technologies in the aeronautics and astronautics fields. At present, there are three kinds of toughening technologies for thermosetting resins: (1) adding second phase materials such as elastomers and rigid particles in a resin; (2) using interpenetrating polymer network technology; (3) introducing a flexible chain segment to molecular structures of a resin (grafting or blocking), or selecting an appropriate crosslinking agent [31]. The method of introducing a flexible molecular chain into the structure of a resin has been widely used in toughening epoxy resins. Lee, et al. [32] modified an epoxy resin with polyethersulfone (PES) and found that the obtained PES/epoxy blend displayed the impact strength, fracture toughness and thermal stability increased by 35%, 11% and 1%, respectively. Ma, et al. [33] synthesized a new hyperbranched poly(urethane-phosphine oxide) (HPUPO) and modified an epoxy resin by blending HPUPO. It was found that the flame retardancy and toughness of the epoxy resin increased with the increase of the modulus and strength of HPUPO, and the surface roughness of the epoxy resin composite also increased. Rosettl et al. [34] studied two PES with different chains as toughening agents of an epoxy resin, and found that the morphology and fracture toughness of the epoxy resin could be adjusted by changing curing conditions. In this paper, in order to expand the functionality of polytriazole resins, we are trying to develop a toughened polytriazloe resin to which poly(ethylene glycol)s with end azide groups was introduced. The effects of the amount and the molecular weight of poly(ethylene glycol)s on the toughness of polytriazole resins are investigated.

## Experimental

### Materials

Poly(ethylene glycol) 400 (A.R), poly(ethylene glycol) 600 (A.R), poly(ethylene glycol) 800 (A.R), thionyl chloride, toluene (A.R), *N,N*-dimethylformamide (DMF) (A.R), dichloromethane(A.R), magnesium sulfate(A.R), acetone were purchased from Shanghai Titan Technology Co., Ltd. 1,1-Bisazidomethyl-4,4′-biphenyl(BAMBP) and *N’,N’,N’,N’*-tetrapropargyl-*p,p’*-diaminodiphenyl methane (TPDDM) were synthesized according to a previously published procedure [16,19]. Toluene was dried and distilled before use.

### Synthesis of azido-terminated poly(ethylene glycol) macromonomers (ATPEG) [35]

#### Synthesis of the chloro-terminated polyethylene glycols

Poly(ethylene glycol)s (80 mmol) and toluene (200 ml) were charged into a 500 ml four-necked flask. After that thionyl chloride (640 mmol) was slowly added in a drop-wise manner within 2 h. This mixture was gradually heated to 80°C and kept at 80°C for 40 h under a machine stirring. After distillation of the toluene under reduced pressure, the liquid was dried at 70 °C under vacuum to afford a transparent dark brown liquid chloro-terminated poly(ethylene glycol)s in yield of 80%~83%. Analysis for chloro-terminated poly(ethylene glycol) 400: ^1^ H-NMR (CDCl_3_, TMS) *δ*:3.76(t,4 H, Cl-CH_2_-), 3.65(m,28 H, -CH_2_-O-). MS-ESI(*m*/*z*): 406(M^+^); chloro-terminated poly(ethylene glycol) 600: ^1^ H-NMR (CDCl_3_, TMS) *δ*: 3.76(t,4 H, Cl-CH_2_-), 3.64(m,48 H, -CH_2_-O-). MS-ESI(*m*/*z*): 626(M^+^); chloro-terminated poly(ethylene glycol) 800: ^1^ H-NMR (CDCl_3_, TMS) *δ*: 3.76(t,4 H, Cl-CH_2_-), 3.65(m,68 H, -CH_2_-O-). MS-ESI(*m*/*z*):847(M^+^). The synthesis procedure is shown in.

**Scheme 1.**
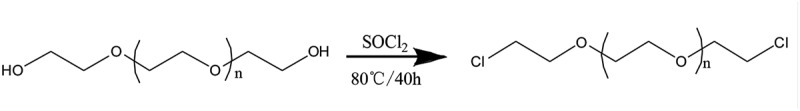
Chlorination route of poly(ethylene glycol)s

#### Synthesis of ATPEG

In a 500 ml three-necked flask with thermometer, mechanical stirrer, and reflux condenser, DMF (350 ml), a chloro-terminated poly(ethylene glycol) (20 mmol), and sodium azide (100 mmol) were added and mixed. This mixture was gradually heated to 80°C and kept at the temperature for 24 h. When cooled to room temperature, the mixture was poured into deionized water (400 ml), and then extracted with dichloromethane (300 ml), and the organic layer was washed with deionized water several times. After the organic layer was dried over MgSO_4_ for 24 h, dichloromethane was distilled off under reduced pressure. After the obtained product was dried in vacuum at 55°C, a pale yellow liquid azide-terminated poly(ethylene glycol) (ATPEG) was gotten in yield of 64%~67%. ATPEG400: ^1^ H-NMR (CDCl_3_, TMS) *δ*: 3.39(t,4 H, N-CH_2_-), 3.67(m,28 H, -CH_2_-O-). MS-ESI(*m*/*z*):420(M^+^). Elemental analysis (%): calculated for C_16_H_32_O_7_N_6_: C, 45.71; H, 7.62; N, 20.00; found: C, 46.33; H, 7.08; N, 19.83; ATPEG600: ^1^ H-NMR (CDCl_3_, TMS) *δ*: 3.32(t,4 H, N-CH_2_-), 3.65(m,48 H, -CH_2_-O-). MS-ESI(*m*/*z*): 640 (M^+^). Elemental analysis (%): calculated for C_26_H_52_O_12_N_6_: C, 48.75; H, 8.12; N, 13.13; found: C, 49.78; H, 7.95; N, 11.89; ATPEG800: ^1^ H-NMR (CDCl_3_, TMS) *δ*: 3.32(t,4 H, N-CH_2_-), 3.65(m,68 H, -CH_2_-O-). MS-ESI(*m*/*z*):860 (M^+^). Elemental analysis (%): calculated for C_36_H_72_O_17_N_6_: C, 49.26; H, 8.34; N, 9.87; found: C, 50.23; H, 8.37; N, 9.77. The synthesis procedure is shown in.

**Scheme 2.**
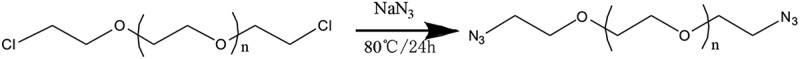
Synthesis route of ATPEGs

### The preparation of EPTA resins

EPTA resins were prepared by the reaction of TPDDM with BAMBP and ATPEGs. The formulation of monomers or/and macromonomers for the synthesis of the resins and the names of the resins are listed in Table 1. The synthesis reaction of EPTA resins is shown in.

**Scheme 3.**
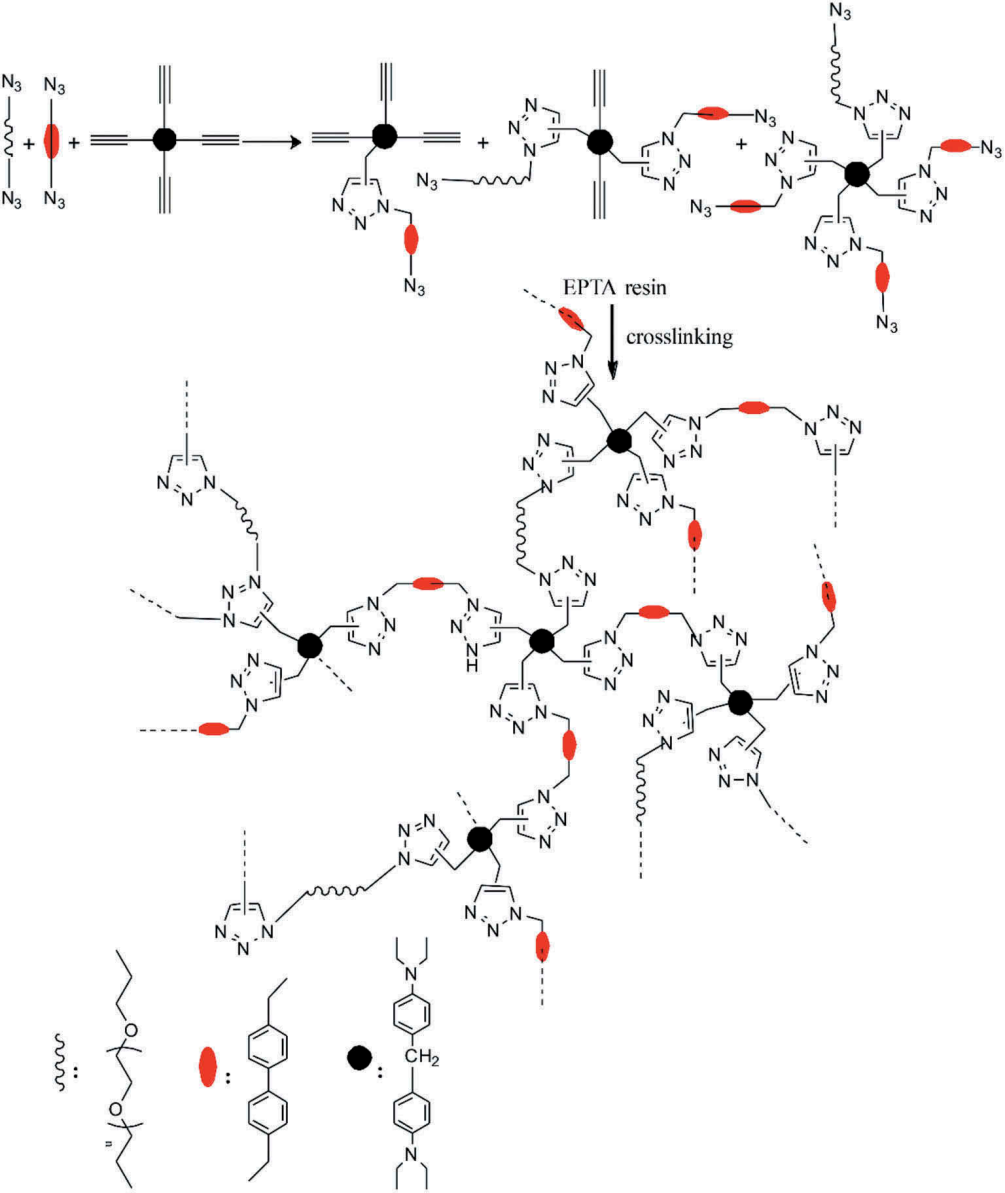
Schematic route of synthesis and curing reactions of EPTA resins

**Table 1.** The formulation of monomers and macromonomers for the synthesis of the extended polytriazole resins

Resin	ATPEG400 (mmol)	ATPEG600 (mmol)	ATPEG800 (mmol)	BAMBP (mmol)	TPDDM (mmol)
PTA	–	–	–	1.00	0.525
EPTA-4-10	0.10	–	–	0.90	0.525
EPTA-4-15	0.15	–	–	0.85	0.525
EPTA-4-20	0.20	–	–	0.80	0.525
EPTA-6-10	–	0.10	–	0.90	0.525
EPTA-6-15	–	0.15	–	0.85	0.525
EPTA-6-20	–	0.20	–	0.80	0.525
EPTA-8-10	–	–	0.10	0.90	0.525
EPTA-8-15	–	–	0.15	0.85	0.525
EPTA-8-20	–	–	0.20	0.80	0.525

TPDDM and BAMBP together with an ATPEG with the molar ratio 1.05:1.00 of alkyne group to azide group ([C ≡ C]/[N_3_]) were mixed in acetone with a solid content of 70% and stirred at 60°C till the mixture became homogeneous. The reaction continued at 60°C for 4 h. The liquid resinwas obtained after acetone was removed by evaporation. The resin prepared from ATPEG400, BAMBP and TPDDM is named as EPTA-4, from ATPEG600, BAMBP and TPDDM as EPTA-6 and from ATPEG800, BAMBP and TPDDM as EPTA-8. Taking EPTA-4-10 resin as an example, it was prepared from TPDDM and azides (10% APTPG400, 90% BAMBP). PTA resin was prepared from BAMBP and TPDDM in the same way. The liquid resins obtained by evaporation were poured into a mold and put into a vacuum oven for removing the residual acetone and embedded air. The resins were cured at 80 °C for 12 h. The obtained resins were post-cured in the following procedure: 120 °C/2 h + 150 °C/2 h + 180 °C/2 h + 210 °C/2 h.

EPTA resins have good solubility due to the introduction of polyethers in the polytriazole structure and dissolve in acetone, THF, acetonitrile, ethyl acetate, chlorinated hydrocarbon solvents and strong polar solvents at room temperature, which is conducive to the subsequent processing and application of resins.

### Instruments and characterization

All NMR spectra were obtained on a Bruker Advance 400 MHz Spectrometer (Bruker, USA) using tetramethylsilane (TMS) as an internal standard in DMSO or CDCl_3_. Electrospray ionization mass spectrometry (ESI-MS) was performed using ESI-high resolution time of flight mass spectrometer (Waters, USA) with m/z range of 50 ~ 4000 Da. FTIR spectrum measurements were carried out on a Nicolet iS10 infrared spectrometer (Madison, USA) in the region of 4000–400 cm^−1^ using KBr pellets. The elemental analysis was performed with Vario EL III elemental analyzer (Elementar, Germany). Differential scanning calorimetry (DSC) analyses were performed with a Q2000 (TA, USA) at a heating rate of 10 °C min^−1^ in a nitrogen atmosphere. Dynamic mechanical analysis (DMA) was carried out on a DMA 1 (Mettler Toledo, Swiss) in the dual cantilever clamp mode under nitrogen at the frequency of 11 Hz with a programmed heating rate of 3 °C min^−1^ from RT to 350 °C. Thermogravimetric analysis (TGA) was conducted on a TGA/DSC 1 (Mettler Toledo, Switzerland) under nitrogen at a heating rate of 10°C min^−1^ from RT to 800 °C and the gas flow rate was 60 ml/min. The flexural performance test was conducted on DDL100 electronic tension machine in accordance with GB/T 2570–1997, and the loading speed was 2 mm/min. The impact performance test was carried out on the Italy CEAST9050 cantilever beam impact testing machine, pendulum energy impact range was 0.55 ~ 22.5 J. No notch impact was tested when the pendulum energy was 4 J with the sample size of 80 mm × 10 mm × 4 mm on reference to GB/T 2571–1995. Hardness was measured by GYZJ-934-1 Barcol impressor. Scanning electron microscopy (SEM) was performed using the S-4800 scanning electron microscope (Hitachi, Japan) with an acceleration voltage of 15 kV.

## Result and discussion

### Curing behavior of EPTA resins

**Figure 1.**
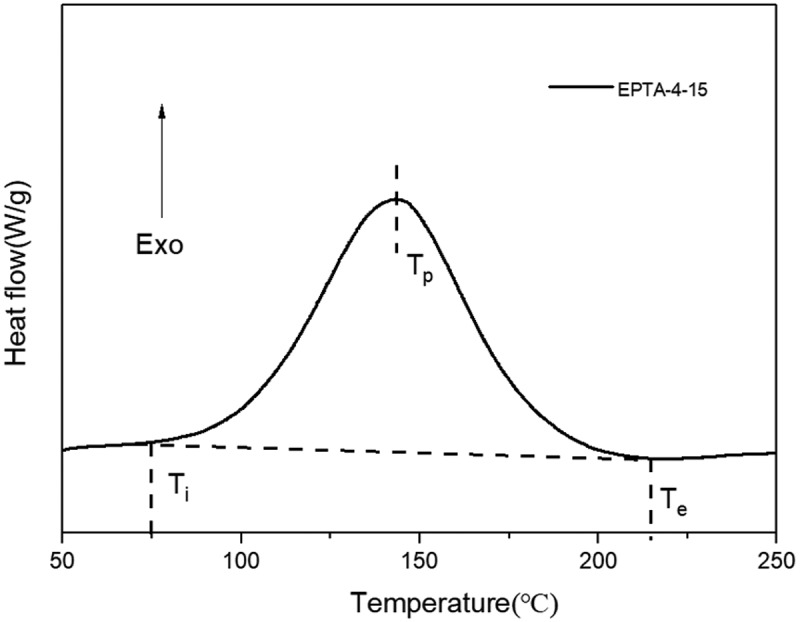
DSC curve of EPTA-4-15 resin

The curing behavior of the EPTA-4-15 was investigated by DSC at a heating rate of 10°C min^−1^ under nitrogen atmosphere and the obtained DSC curve is shown in Figure 1. The DSC curves of other resins are in the SI (Figure S1-S9). Specific values are listed in Table 2. It can be seen that the exothermic peak of the resin appears between 70 ~ 220°C, which is due to an exothermic reaction of 1,3-dipolar cycloaddition between azide and alkyne groups.

**Table 2.** DSC analysis results of EPTA resins

Resin	T_i_(°C)	T_p_(°C)	T_e_(°C)	*∆H*(J/g)
PTA	79	142	200	858
EPTA-4-10	78	148	211	797
EPTA-4-15	74	143	209	878
EPTA-4-20	80	144	208	869
EPTA-6-10	77	144	210	673
EPTA-6-15	74	148	210	801
EPTA-6-20	75	146	209	701
EPTA-8-10	75	145	213	719
EPTA-8-15	78	147	211	783
EPTA-8-20	76	147	210	687

The DSC curves show that the initial curing temperature of EPTA resins is around 80°C, the peak temperature of exothermic peak is around 145°C, and the end temperature is around 200°C. The detailed structures of three EPTA resins are different, but their initial curing temperatures and peak temperatures are very close, indicating that the ‘Click’ reaction of alkyne and azide is less affected by the connected structures. The exothermic heat *∆H* of the resins is relatively high (> 670 J/g), so in order to avoid explosive polymerization during the curing process, the initial curing reaction temperature should be determined to be around T_i_.

**Figure 2.**
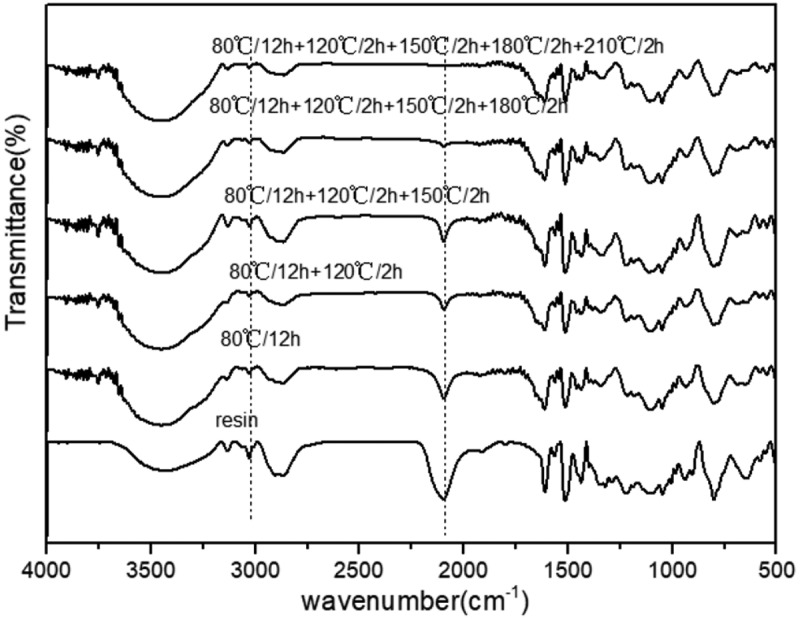
FTIR spectra of EPTA-4-15 resin at different curing stages

The curing process of EPTA-4-15 resin is studied by FTIR analysis. The absorption of the characteristic functional groups of the EPTA-4 resin changes at different curing stages in the FTIR spectra during the process of polymerization as shown in Figure 2. It can be seen from Figure 2 that the characteristic absorption peak of –N_3_ and –C≡C– near 2095 cm^−1^ gradually decreases with the curing process, and the stretching vibration peak of carbon-hydrogen bonds on triazole rings appears near 3034 cm^−1^, indicating that the polymerization reaction of –N_3_ and –C≡C– occurs. With the increase in polymerization temperature, the characteristic absorption peak of –N_3_ and –C≡C– at 2095 cm^−1^ gradually weakens and completely disappears at 210 °C, indicating that the curing of EPTA-4-15 resin is completed. The results of FTIR characterization of EPTA-6 resins and EPTA-8 resins are similar(see SI Fiure S10-S11). FTIR characterization also shows that the resins finish the curing reactions through the curing procedure of 80 °C/12 h + 120 °C/2 h + 150 °C/2 h + 180 °C/2 h + 210 °C/2 h.

### Effect of the amount of ATPEG on mechanical properties of cured EPTA resins

The mechanical properties of cured EPTA resins are listed in Table 3. It can be seen that the addition of ATPEG significantly improves impact properties of the produced resins compared to PTA resin. The toughness of a resin is a characterization of the ability of the resin to absorb and dissipate energy when the resin is subjected to a sudden applied load. Generally speaking, a resin with a flexible (loose) crosslinked chain could absorb and dissipate energy more efficiently than that with a rigid chain [36–38]. Also, Proper cross-linking can effectively increase the connection between molecular chains while overly crosslinked structure leads to brittleness [39,40]. ATPEG has long and flexible polyether chains. The introduction of the polyether into the resin can increase the flexibility of the chain segments while its long chain segments can appropriately reduce the crosslinking degree. Therefore, EPTA resins display higher impact strength.

**Table 3.** Mechanical properties of cured EPTA resins

Cured resin	Impact strength (KJ/m^2^)	Flexural strength (MPa)	Flexural modulus (GPa)	Hardness (HBa)
PTA	38.0 ± 2.8	128.0 ± 2.8	2.55 ± 0.04	32 ± 1.6
EPTA-4-10	46.3 ± 4.4	113.1 ± 2.0	2.57 ± 0.02	31 ± 1.9
EPTA-4-15	64.0 ± 3.3	108.4 ± 2.3	2.57 ± 0.01	26 ± 1.2
EPTA-4-20	70.3 ± 3.0	104.5 ± 2.7	2.60 ± 0.05	23 ± 2.2
EPTA-6-10	54.9 ± 4.3	105.5 ± 3.2	2.72 ± 0.07	21 ± 1.6
EPTA-6-15	75.0 ± 4.4	102.6 ± 2.1	2.56 ± 0.06	17 ± 1.0
EPTA-6-20	79.6 ± 3.1	83.99 ± 1.9	2.24 ± 0.04	13 ± 2.3
EPTA-8-10	56.3 ± 2.5	97.80 ± 1.2	2.63 ± 0.01	21 ± 1.9
EPTA-8-15	59.1 ± 3.1	96.01 ± 2.6	2.41 ± 0.06	16 ± 1.3
EPTA-8-20	62.1 ± 3.3	84.27 ± 0.6	2.31 ± 0.02	10 ± 2.7

**Figure 3.**
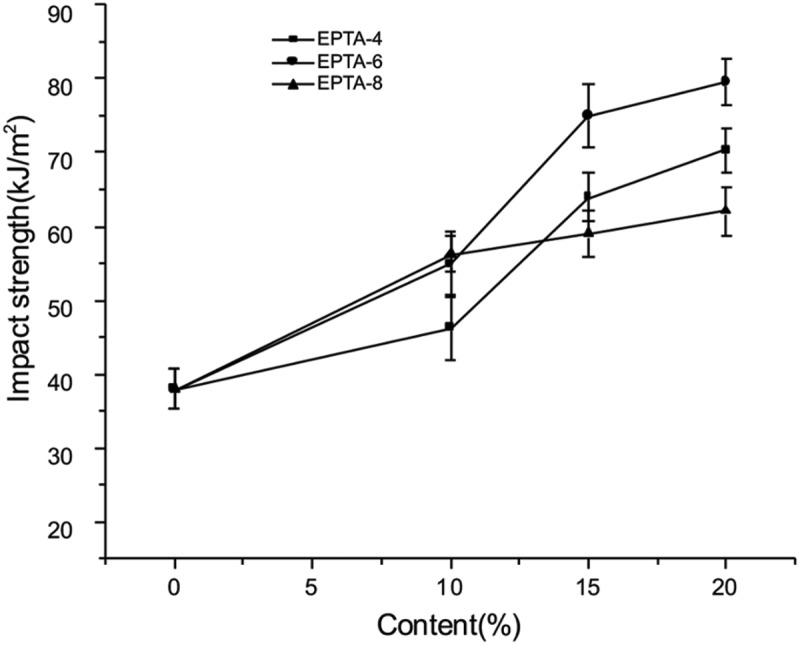
The relationship between the amount of ATPEGs and the impact strength of cured EPTA resins

Figure 3 shows the relationship between the amount of ATPEGs added and the impact strength of cured EPTA resins. As shown in the Figure, when the molecular weight of ATPEG is the same, with the amount of ATPEG increasing, the impact strength of the cured EPTA resin increases. And when the molecular weight of ATPEG is 400 and 600, the impact strength of EPTA resin increases significantly with the increase of the added amount. For example, when adding 10%, 15%, and 20% ATPEG600, the impact strength of EPTA resins increased by 44.5%, 97.4%, and 100.8% compared with PTA resin, respectively. This is because when ATPEG is added to the polytriazole resin as a toughing agent, the azide group of the polyether reacts with the tetraalkyne to form a triazole ring and a flexible polyether chain segment connects into the polymer network. The higher the proportion of polyether is, i.e., the more the polyether segment in the resin structure is, the higher the flexibility of the produced resin. Thereby, the resins with high ATPEG addition amount have high impact strength.

**Figure 4.**
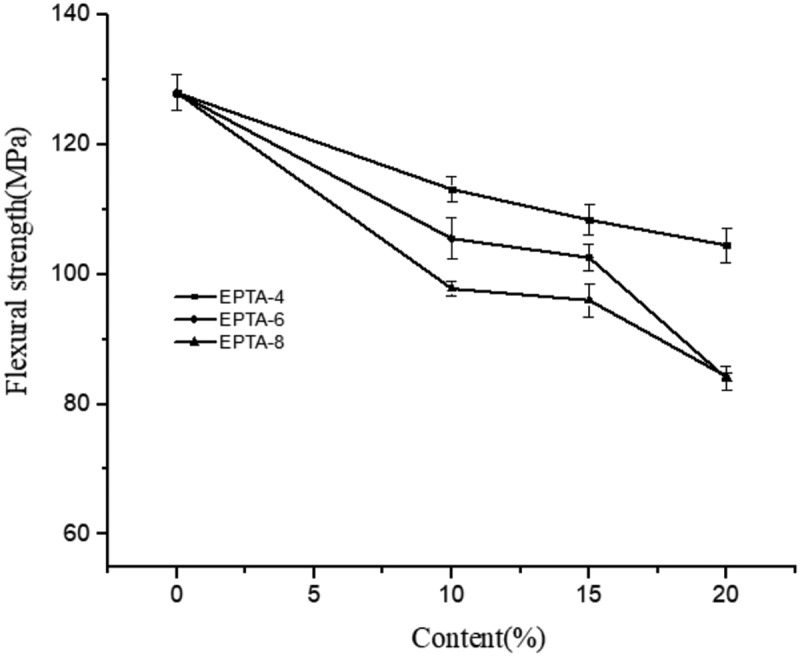
The relationship between the amount of ATPEGs and the flexural strength of cured EPTA resins

Figure 4 shows the relationship between the amount of ATPEG added in EPTA resin and the flexural strength of cured EPTA resins. Obviously, the flexural strength of EPTA resins decreases to some extent compared with that of PTA resin. For polyethers with the same molecular weight, the flexural strength of the cured EPTA resin decreases with the increase in the amount of ATPEGs. This is because as the polyether segment in the crosslinked network increases, the rigidity of chain segment decreases and the crosslink density of the resin also decreases, resulting in a reduction in the flexural strength of the resin.

The hardness of cured resins was measured with a Barcol impressor and shown in Table 3. The hardness of cured EPTA resins decreases with the increase in the amount of ATPEGs and molecular weight of ATPEGs. The hardness of the EPTA resin has a 70% decrease at most, compared with that of the PTA resin. The decrease of hardness of EPTA resins is consistent with the change of flexural strength, which further verify the increase of flexibility and decrease of crosslinking degree of EPTA resins.

### Effect of the molecular weight of ATPEG on mechanical properties of cured EPTA resins

The molecular weight between crosslinked points affects the properties of the resin. When the molecular weight is high, the distance between crosslinking points is long, leading to lower crosslinking degree. Thereby, the resin with high molecular weight flexible chain would exhibit high impact strength and low flexural strength and hardness.

**Figure 5.**
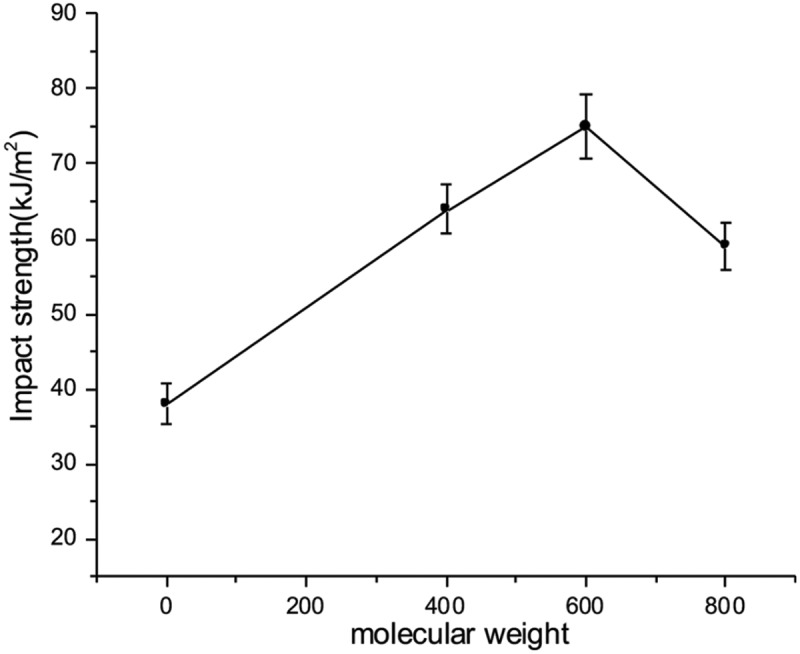
The relationship between the impact strength of cured EPTA resins and the molecular weight of ATPEGs (EPTA-i-15, i = 4, 6, 8)

**Figure 6.**
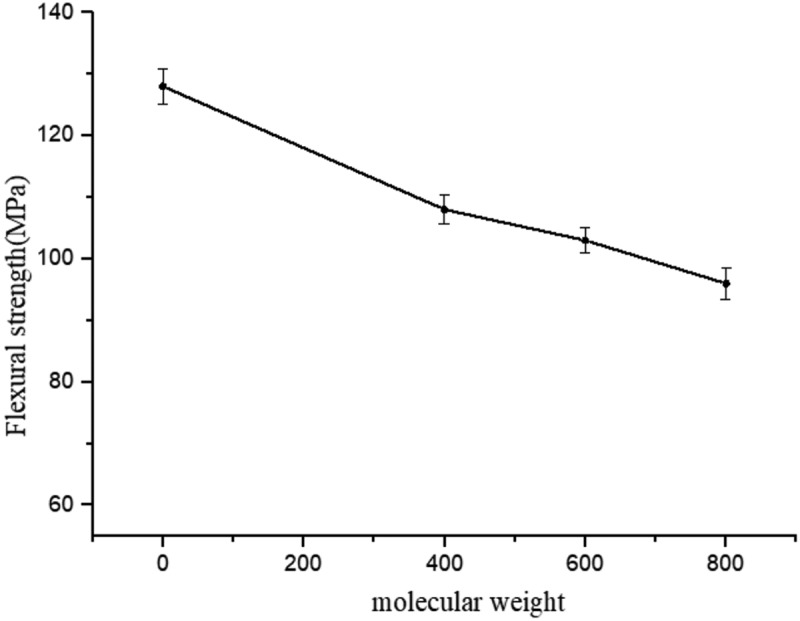
The relationship between the flexural properties of cured EPTA resins and the molecular weight of ATPEGs (EPTA-i-15, i = 4, 6, 8)

The impact and flexural properties of EPTA resins toughened with various molecular weight of ATPEGs are shown in Figures 5 and Figures 6. As shown in Figure 5, the impact strength of EPTA resins is related to the molecular weight of ATPEGs. When the amount of ATPEGs is 15%, as the molecular weight of ATPEGs increases, the impact strength of EPTA resins first increases and then decreases. The impact strength of the resin reaches the highest value when the molecular weight of polyether is 600. When the molecular weight of polyether arrives at 800, the impact strength of the EPTA-8 resin decreases. The possible reason is that ATPEG800 with higher molecular weight easily aggregates together and even phase separation may happen in the resin. The further investigation will be undertaken.

Figure 6 shows the relationship between the molecular weight of ATPEGs and flexural strength of EPTA resin. It can be seen that for the same amount of ATPEG, the flexural strength of cured EPTA resins shows a decreasing trend with the increase of molecular weight of ATPEGs. When the molecular weight of flexible polyether chain segments increases, the distance between the crosslinking points increases to result in decrease of the crosslinking degree, at the same time, the rigidity of the crosslinked network decreases. Therefore, the flexural strength of resin decreases with the increase in the molecular weight of ATPEGs.

### Microstructure analysis of cured EPTA resins

**Figure 7.**
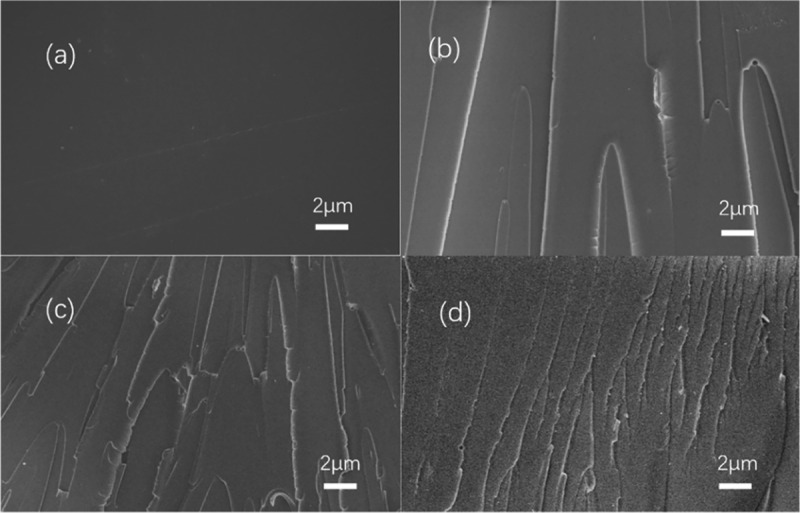
SEM images of PTA resin and EPTA resins

The microstructure of the resins is particularly important for their properties. The fracture morphology of resins observed by scanning electron microscopy can provide necessary information for the properties and modification of the resin. Figure 7 shows the SEM images of impact fracture sections of PTA resin and EPTA resins. It can be seen from Figure 7(a) that the fracture surface of the PTA resin is relatively smooth and there are a few shallow stress streaks. These are the characters for resins with poor toughness. The impact section of the EPTA resins (Figure 7(b–d)) can be seen to be uneven. With the molecular weight of the polyether increased to 800, the fracture becomes less steep, which shows the tough morphology. This is because EPTA resin added with ATPEGs has better toughness. When high-speed impact is applied, more energy can be absorbed and crack propagation is hindered, effectively dispersing the phenomenon of stress concentration.

(a) PTA; (b)EPTA-4-20; (c)EPTA-6-20; (d)EPTA-8-20

### Thermal properties of cured EPTA resins

**Figure 8.**
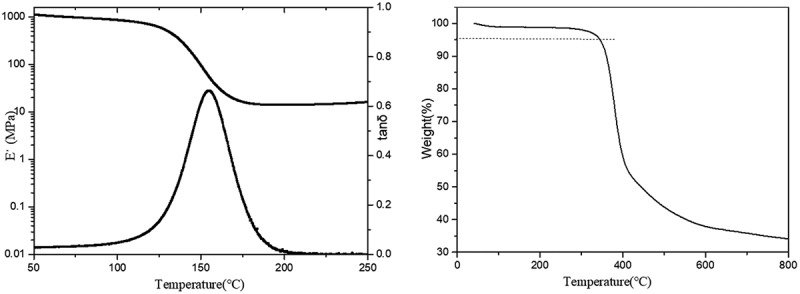
DMA and TGA curves of cured EPTA-6-15 resin

The DMA analyses of various cured EPTA resins were conducted and typical DMA curves are shown in Figure 8. The DMA curves of other resins are in the SI (Figure S12-S20). The analysis results are tabulated in Table 4. The results show that the glass transition temperature (T_g_) of the EPTA resins is significantly lower than that of PTA resin. T_g_ of the toughened resin system decreases by 20°C to 30°C for every 5% increase in the amount of ATPEG. For resin systems with the same amount of ATPEG addition, the T_g_ of EPTA resins decreases with the increase of polyether molecular weight. With the increase of the amount and molecular weight of ATPEGs, the network has more flexible chain segment and looser nets (lower crosslinking degree) [38]. As a result, the glass transition temperature of the EPTA resins reduces.

**Table 4.** Thermal properties of cured EPTA resins

Cured resin	T_g_(°C)	T_d5_(°C)	Y_800_(%)
PTA	218	353	34.4
EPTA-4-10	190	341	46.7
EPTA-4-15	169	350	38.2
EPTA-4-20	157	331	37.8
EPTA-6-10	181	347	53.1
EPTA-6-15	155	345	45.8
EPTA-6-20	122	341	40.1
EPTA-8-10	157	339	35.7
EPTA-8-15	129	347	39.3
EPTA-8-20	107	345	32.9

The thermogravimetric analysis of the cured EPTA resins was carried out under nitrogen. The TGA curve for EPTA-4-10 resin is shown in Figure 8. The TGA curves of other resins are in the SI (Figure S12-S20). The analysis results are listed in Table 4. As shown in Table 4, there is little difference for T_d5_ among EPTA resins and T_d5_ is located around 340°C. A large number of rigid structures of a benzene ring and a triazole ring with high crosslinked degree by thermal crosslinking reactions in the curing process contribute excellent thermal stability of the resins. When the temperature is above 365°C, the resin decomposes rapidly. The thermal degradations of EPTA resins are mainly due to the thermal decomposition of the triazole ring and the thermal cleavage of the CH_2_-N bond [21].

## Conclusion

1. Three azide-terminated poly(ethylene glycol) macromonomers (ATPEG400, ATPEG600 and ATPEG800) and related EPTA resins with various amount of ATPEGs were synthesized and characterized. The EPTA resins can be cured at 80 °C, and then post-cured through the procedure of 120 °C/2 h + 150 °C/2 h + 180 °C/2 h + 210 °C/2 h.
2. Molecular weight and amount of ATPEGs have remarkable influence on the thermal and mechanical properties of EPTA resins. The impact strength of EPTA resins increases with the increase in the amount and molecular weight of ATPEGs. When the amount of ATPEG is 15% and 20%, the impact strength of EPTA resin increases first and then decreases with the increase of molecular weight of ATPEG and reaches the highest value when the molecular weight of polyether is 600. The flexural strength and heat resistance of EPTA resins decrease with the increase of addition amount and molecular weight of ATPEGs. The highly toughed EPTA resins could be obtained by control of the amount and molecular weight of polyethers.
3. ATPEG600 shows better toughening effect than ATPEG800. EPTA-6-15 resin demonstrates good comprehensive properties. The impact strength and flexural strength reach 75 kJ/m^2^ and 103 MPa while T_g_ and T_d5_ are 155 °C and 345 °C respectively.
